# Investigation of differentially expressed genes related to cellular senescence between high-risk and non-high-risk groups in neuroblastoma

**DOI:** 10.3389/fcell.2024.1421673

**Published:** 2024-07-29

**Authors:** Xingyu Zhou, Yuying Wu, Lan Qin, Miao Zeng, Mingying Zhang, Jun Zhang

**Affiliations:** ^1^ Ministry of Education Key Laboratory of Child Development and Disorders, Chongqing, China; ^2^ National Clinical Research Center for Child Health and Disorders, Chongqing, China; ^3^ China International Science and Technology Cooperation Base of Child Development and Critical Disorders, Chongqing, China; ^4^ Department of Surgical Oncology, Children’s Hospital of Chongqing Medical University, Chongqing, China; ^5^ Chongqing Key Laboratory of Pediatrics, Chongqing, China

**Keywords:** cellular senescence, DEGs, SRG, prognostic model, risk stratification, neuroblastoma

## Abstract

**Object:**

This study aims to identify differentially expressed genes (DEGs) between high-risk and non-high-risk groups in neuroblastoma (NB), construct a prognostic model, and establish a risk score formula.

**Materials and methods:**

The NB dataset GSE49710 (n = 498) from the GEO database served as the training cohort to select DEGs between high-risk and non-high-risk NB groups. Cellular senescence-related genes were obtained from the Aging Atlas database. Intersection genes from both datasets were identified as key genes of cellular senescence-related genes (SRGs). A prognostic model was constructed using Univariate Cox regression analysis and the Lasso algorithm with SRGs. Validation was performed using the E-MTAB-8248 cohort (n = 223). The expression levels of AURKA and CENPA were evaluated via RT-qPCR in two clinical NB sample groups.

**Results:**

Eight SRGs were identified, and a prognostic model comprising five genes related to cellular senescence was constructed. AURKA and CENPA showed significant expression in clinical samples and were closely associated with cellular senescence.

**Conclusion:**

The prognostic model consisted with five cellular senescence related genes effectively predicts the prognosis of NB patients. AURKA and CENPA represent promising targets in NB for predicting cellular senescence, offering potential insights for NB therapy.

## 1 Introduction

Neuroblastoma (NB) stands as the most common extracranial solid tumor in children (Matthay et al., 2016). While the adoption of multimodal therapy including surgery, radiation, and aggressive combination chemotherapy has improved the outcomes for many children with high-risk NB, the overall survival rate for children in the high-risk group remains below 50% (Maris, 2010). Therefore, the treatment of children with high-risk NB remains a challenge.

Cellular senescence, characterized by stress-induced cell cycle arrest, occurs across diverse cell types (Muñoz-Espín and Serrano, 2014; Pérez-Mancera et al., 2014). During tumor development and progression, cellular senescence can limit cell proliferation and serve as a mechanism to halt tumor growth (Muñoz-Espín and Serrano, 2014; Zanotti et al., 2022). Establishing a prognostic model based on cellular senescence-related genes can help evaluate the relationship between prognosis and cellular senescence in children with NB. Prognostic outcomes differ significantly between high-risk and non-high-risk NB groups. Investigating the disparities in cellular senescence-related signatures may provide insights into the progression, recurrence, drug resistance, or prognosis of high-risk NB, potentially leading to insights for personalized treatment approaches.

Our study entailed the screening of pivotal genes linked to cellular senescence, the development of a prognostic model based on cellular senescence, and the establishment of a risk score formula. This systematic investigation into the interplay between cellular senescence and prognosis in NB elucidates their potential as prognostic biomarkers.

## 2 Materials and methods

### 2.1 Data collection

In this study, we utilized the Gene Expression Omnibus (GEO) database (http://www.ncbi.nlm.nih.gov/geo/) to retrieve the gene expression profile of NB from dataset GSE49710 (Zhong et al., 2018) as our training cohort. Similarly, the ArrayExpress database (https://www.ebi.ac.uk/biostudies/arrayexpress) provided the gene expression profile from dataset E-MTAB-8248 (Wang et al., 2020) for our validation cohort. The training cohort, GSE49710, comprises 498 neuroblastoma samples, encompassing critical clinical information such as gender, age at diagnosis, INSS stage, MYCN status, risk stratification, and patient survival status. The validation cohort, E-MTAB-8248, includes 223 NB samples, with detailed clinical data including age, INSS stage, MYCN status, risk stratification, and survival status. In our analysis, NB samples were classified into high-risk and non-high-risk groups based on their risk stratification. These details are summarized in Supplementary Table S1.

The Aging Atlas website (https://ngdc.cncb.ac.cn/aging/index) offers a comprehensive range of omics services related to aging research. In our study, we utilized this resource to identify genes associated with cellular senescence. These genes are detailed in Supplementary Table S2.

### 2.2 Extraction of the differentially expressed genes (DEGs) and cellular senescence-related genes (SRGs)

In this study, the limma package (Ritchie et al., 2015) in R software (version 4.2.3) was utilized to identify differentially expressed genes (DEGs) between the high-risk and non-high-risk groups of NB in the training cohort. The analysis results are detailed in Supplementary Table S3 and illustrated using volcano plot. The criteria for significant differences were set at an adjusted *p*-value (adj.P) of less than 0.05 and an absolute log2 fold change (|log2FC|) greater than 1. Upregulated and downregulated genes are reported relative to the non-high-risk group. Furthermore, the Bioinformatics & Evolutionary Genomics website (https://bioinformatics.psb.ugent.be) served as an online tool to identify overlaps between DEGs and genes associated with cellular senescence. These findings are summarized in Supplementary Table S4 and depicted in a Venn diagram. In conclusion, eight senescence-related genes (SRGs) were identified through these methods. The overall survival (OS) associated with these SRGs was analyzed using Kaplan-Meier survival curves in GraphPad Prism version 9.5.1.

### 2.3 Construction of the cellular senescence-related signatures (SRS)

Univariate Cox regression analysis was employed to select senescence-related genes (SRGs) using SPSS version 23, detailed in Supplementary Table S5. We applied the LASSO Cox regression (Tibshirani, 1997) method via the “glmnet” package in R to minimize the number of genes and establish a cellular senescence-related risk score formula. This formula calculates the risk score as a linear combination of selected gene expressions weighted by their respective coefficients, optimized through 10-fold cross-validation:

$$SRS=\sum_{i=1}^{N}\left(\mathrm{Exp}i*\text{Coe}i\right)$$

Patients in the training cohort were stratified into high- and low-score groups based on the median SRS. The prognostic utility of the SRS was assessed using time-dependent receiver operating characteristic (ROC) curves, with external validation performed on the E-MTAB-8248 cohort.

### 2.4 Statistics analysis

Data analysis and graph generation were performed using R software (version R4.2.3) R Project, SPSS Statistics V23.0, and GraphPad Prism 9.5.1. Kaplan-Meier survival curves illustrating OS were generated in GraphPad Prism 9.5.1. Time-dependent ROC curves for 1-year, 3-year, and 5-year survival rates were plotted using the “time ROC” R package to evaluate the predictive efficacy of SRS scores. All statistical tests were bilateral, with a significance threshold set at *p* < 0.05.

### 2.5 Functional analysis

Functional and pathway enrichment analyses of SRGs were conducted using the Kyoto Encyclopedia of Genes and Genome (KEGG) and Gene Ontology (GO). The results were visualized to highlight significant enrichment pathways.

### 2.6 Clinical samples and real-time fluorescence quantitative polymerase chain reaction (RT-qPCR)

Sixteen children with NB, treated surgically at the Department of Oncology, Children’s Hospital of Chongqing Medical University between 2021 and 2024, were selected for this study. Half were classified as high-risk and the other half as non-high-risk. Tumor tissues, collected post-surgery, were stored at −80°C. Only patients who had not received preoperative chemotherapy or radiotherapy were included. The study was approved by the Ethics Committee of the Children’s Hospital Affiliated to Chongqing Medical University. Total RNA was extracted using a liquid nitrogen grinding method, followed by reverse transcription. RT-qPCR was performed according to standard protocols, with conditions of 95°C for 3 min, followed by 45 cycles of denaturation at 95°C for 5 s, annealing at 60°C, and extension for 34 s. Gene expression levels were normalized against β-ACTIN using the 2^−ΔΔCt method, with primers sourced from the Primer Bank (https://pga.mgh.harvard.edu/primerbank/). The primer sequence is shown in Table 1.

**TABLE 1:** The primer sequence.

Gene name	Primer sequence
β-ACTIN	F-primer:5’-CCTGGCACCCAGCACAAT-3’
	R-primer:5’-GGGCCGGACTCGTCATAC-3’
AURKA	F-primer:5’-GAGGTCCAAAACGTGTTCTCG-3’
	R-primer:5’-ACAGGATGAGGTACACTGGTTG-3’
CENPA	F-primer:5’- GAC​GCC​TAT​CTC​CTC​ACC​TTA-3’
	R-primer:5’- GTT​GCA​CAT​CCT​TTG​GGA​AGA-3’

## 3 Results

### 3.1 Screening of SRGs in NB cells

**FIGURE 1:**
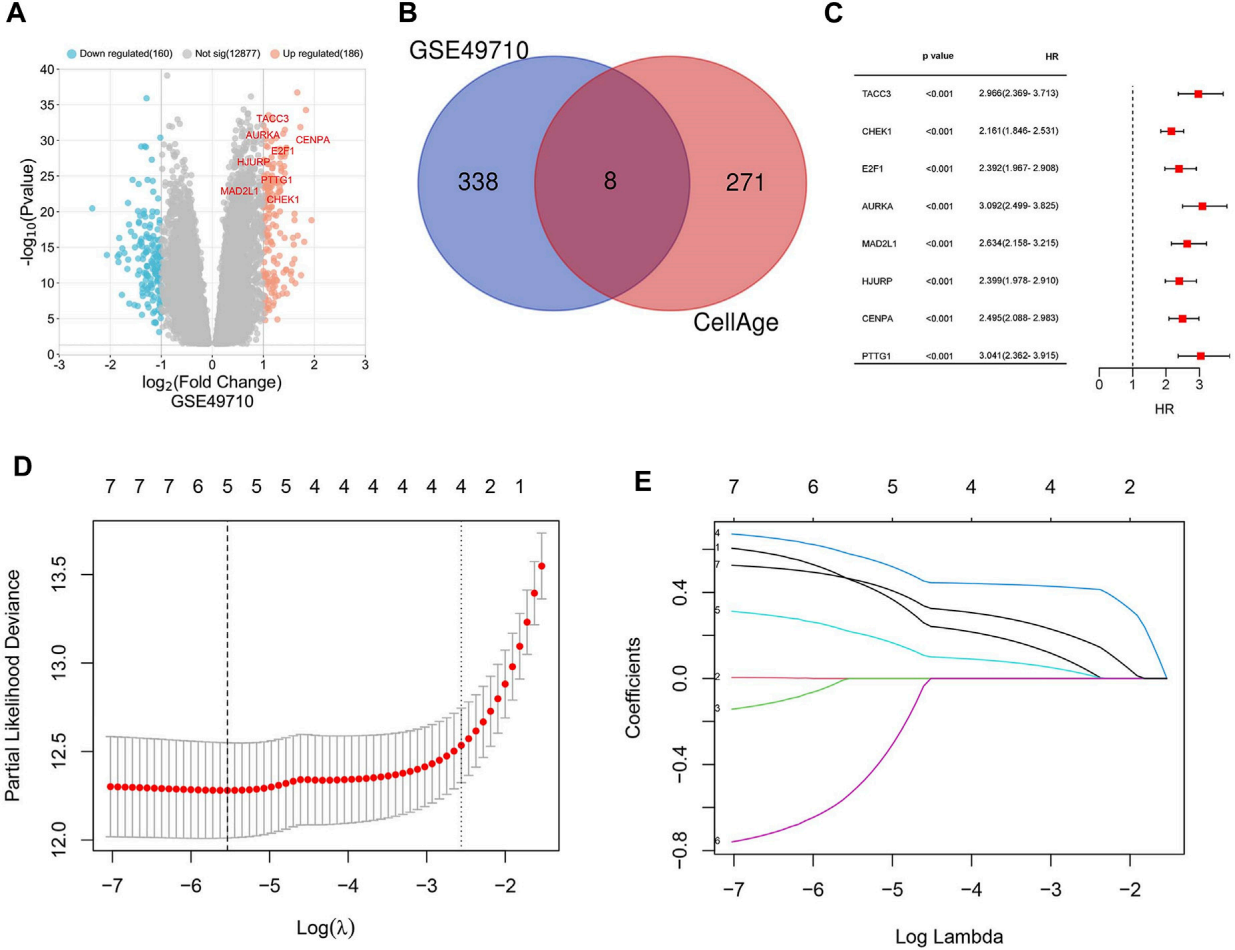
Identification of differentially expressed genes (DEGs) and cellular senescence-related genes (SRGs). **(A)** Volcano plot depicting DEGs in dataset GSE49710. **(B)** Venn diagram showing the overlap between DEGs and genes associated with cellular senescence. **(C)** Forest plot of the univariate Cox regression analysis identifying 8 SRGs. **(D)** Lasso coefficient curves for five key SRGs: AURKA, MAD2L1, HJURP, CENPA, TACC3. **(E)** Ten-fold cross-validation for tuning parameter optimization in Lasso models.

**FIGURE 2:**
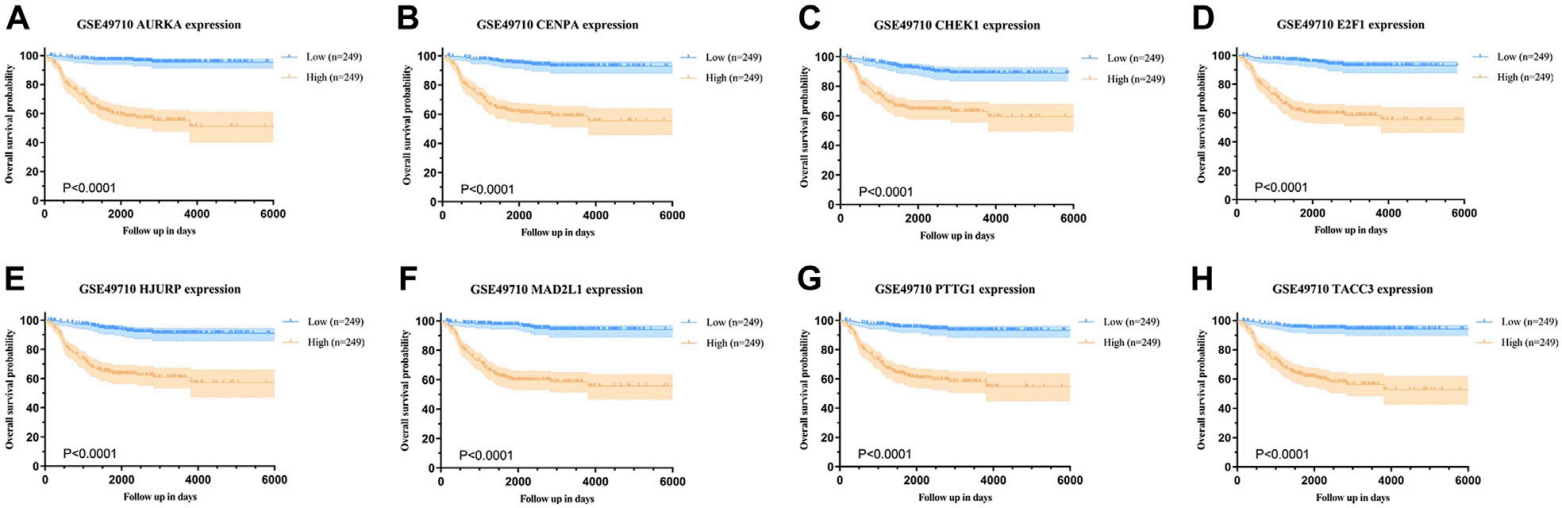
Overall survival analysis based on SRG expression in neuroblastoma (NB). **(A–D)** Kaplan-Meier survival curves for AURKA, CENPA, CHEK1, and E2F1, comparing high vs low expression groups in NB. **(E–H)** Kaplan-Meier survival curves for HJURP, MAD2L1, PTTG1, and TACC3, comparing high vs low expression groups in NB.

Background correction and normalization were applied to the NB dataset GSE49710 using the “limma” package in R. This analysis identified 346 DEGs, with 186 upregulated and 160 downregulated, as illustrated in the volcano plot (Figure 1A). Further analysis targeting 279 senescence-associated genes revealed an intersection with DEGs through the Bioinformatics & Evolutionary Genomics platform, identifying eight significant SRGs: TACC3, CHEK1, E2F1, AURKA, MAD2L1, HJURP, CENPA, and PTTG1 (Figure 1B). Kaplan-Meier survival curves for these genes demonstrated varied impacts on OS (Figures 2A–H).

Pathway and functional enrichment analyses using KEGG and GO highlighted significant involvement of these SRGs in critical biological processes such as the mitotic cell cycle, cell differentiation, and specific nucleosome assembly related to CENP-A. The molecular functions predominantly associated with these genes included protein and DNA binding. These SRGs also featured in pathways relevant to cell cycle regulation, response to Human T-cell leukemia virus 1, and oocyte meiosis, detailed in Supplementary Figure S1.

### 3.2 Construction of a prognostic model for cellular senescence-related signatures (SRS)

**FIGURE 3:**
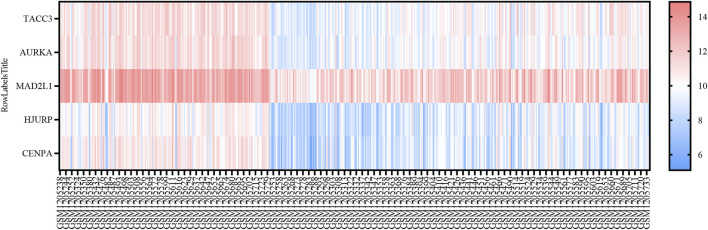
Heatmap Representation of SRGs in GSE49710. Heatmap showing the expression levels of five SRGs in GSE49710, ranging from blue (low expression) to red (high expression).

Univariate Cox regression analysis was applied to eight SRGs to determine their association with OS. The results, illustrated in the forest plot (Figure 1C), indicated significant correlations for all genes (*p* < 0.05, Supplementary Table S5). To refine the model, the LASSO algorithm was utilized, resulting in a five-gene model derived from the optimal λ value, depicted in Figures 1D, E. The heatmap of these genes is shown in Figure 3.

Based on the expression levels of these five genes in NB patients, the following risk scoring formula was established:

SRS = 0.5782* AURKA expression value +0.2177* MAD2L1 expression value -0.5369* HJURP expression value +0.4630* CENPA expression value +0.4594* TACC3 expression value.

**FIGURE 4:**
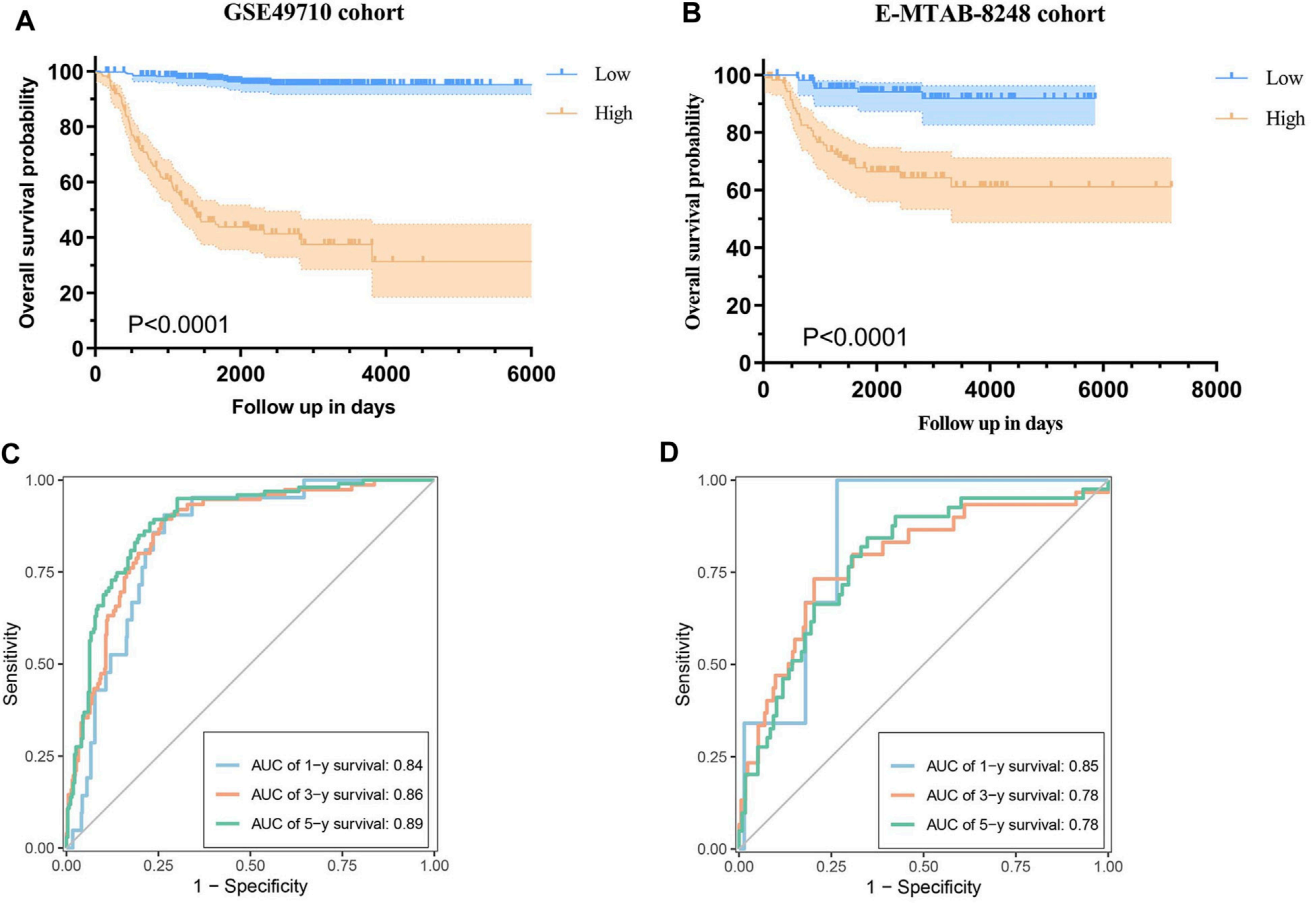
Validation of the Prognostic Model. **(A)** Kaplan-Meier curves illustrating overall survival (OS) in the GSE49710 cohort based on the risk score. **(B)** Time-dependent ROC curve analysis for 1-year, 3-year, and 5-year OS in the GSE49710 cohort. **(C)** Kaplan-Meier curves illustrating OS in the E-MTAB-8248 cohort based on the risk score. **(D)** Time-dependent ROC curve analysis for 1-year, 3-year, and 5-year OS in the E-MTAB-8248 cohort.

This scoring formula was utilized to calculate the risk score for each patient in the training cohort GSE49710, subsequently categorizing them into low and high-score groups based on the median risk score. Kaplan-Meier survival analysis revealed a significantly shorter OS in the high-score group compared to the low-score group (Figure 4A, log-rank *p < 0.0001*). Furthermore, the model demonstrated excellent predictive performance with area under the curve (AUC) values of 0.84, 0.86, and 0.89 for 1-year, 3-year, and 5-year OS respectively, according to time-dependent ROC analysis (Figure 4C).

### 3.3 Validation of the prognostic model in an independent cohort

The prognostic model was evaluated within an independent validation cohort (E-MTAB-8248) by calculating the risk score for each patient using the established formula. Patients were stratified into low- and high-score groups based on the median risk score. Subsequently, survival curves and time-dependent ROC curves were generated to assess the model’s performance. Kaplan-Meier survival analysis revealed that patients in the high-risk group experienced significantly shorter OS compared to those in the low-risk group (Figure 4B, log-rank *p < 0.0001*). The time-dependent ROC analysis indicated that the AUC values for predicting 1-year, 3-year, and 5-year OS was 0.85, 0.78, and 0.78, respectively (Figure 4D).

### 3.4 Validation of target genes by RT-qPCR

**FIGURE 5:**
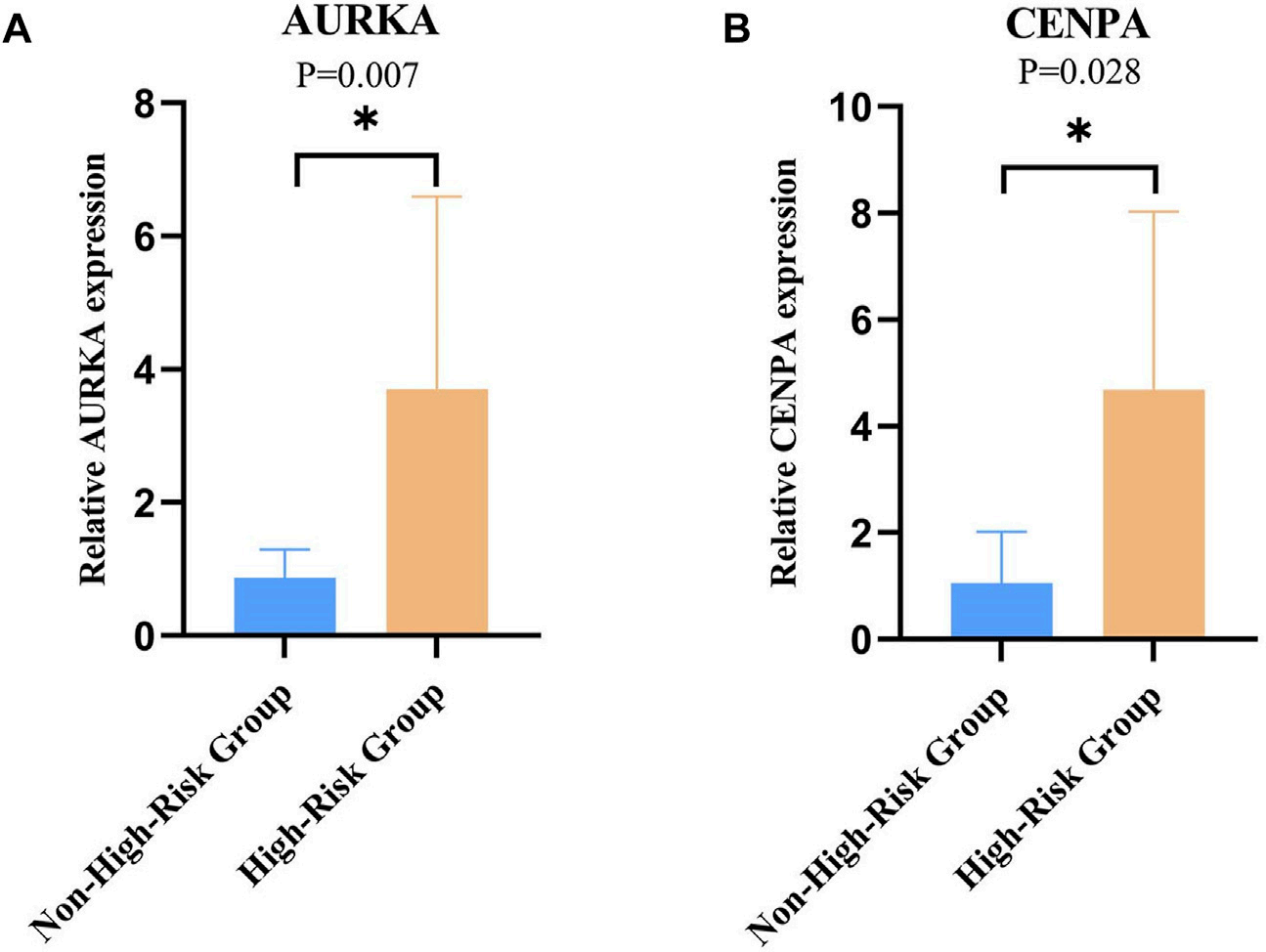
Relative Expression of AURKA and CENPA in Clinical Samples. **(A)** Relative expression of AURKA in high-risk vs non-high-risk groups of clinical samples. **(B)** Relative expression of CENPA in high-risk vs non-high-risk groups of clinical samples, with statistical significance indicated (*p < 0.05).

To clinically validate the key genes identified in our prognostic model, we focused on AURKA and CENPA due to their high alteration frequency in NB. A total of 16 NB samples were analyzed for the CENPA gene expression, with equal representation from both high-risk (n = 8) and non-high-risk (n = 8) groups. Similarly, 14 NB samples were evaluated for AURKA gene expression, comprising seven samples each from high-risk and non-high-risk groups. The RT-qPCR results demonstrated statistically significant differences in the expression levels of both genes between the groups (*P < 0.05*, Figure 5). Specifically, the expression level of AURKA was significantly elevated in the high-risk group compared to the non-high-risk group (*P* = 0.007, Figure 5A), and a similar pattern was observed for CENPA, with higher expression in the high-risk group (*P* = 0.028, Figure 5B).

## 4 Discussion

In cellular biology, the inevitable transition from cell growth to cellular senescence and subsequent death is a fundamental metabolic principle, with cell lifespan varying by types (Sender and Milo, 2021). Conventionally, cellular senescence has been recognized as a mechanism that curtails cellular proliferation and potentially thwarts tumor development (Muñoz-Espín and Serrano, 2014). Currently, most anticancer drugs induce cellular senescence by disrupting replication, blocking nucleic acid synthesis, or interfering with the G1/S phase transition, thereby leveraging anti-tumor effects, a strategy known as pro-senescence therapy for cancer (Liang et al., 2019; Poratti and Marzaro, 2019).

The induction of cellular senescence is typically related to the p53/p16 cell cycle inhibition pathway. Various stressors or damage factors activate the tumor suppressor RB (retinoblastoma-associated protein, pRB, RB1) through the p53/p16 signaling pathway, leading to cellular senescence (Paez-Ribes et al., 2019; Domen et al., 2022). AURKA, a member of serine/threonine kinase family (Aurora kinase) (Du et al., 2021) is a key regulatory component involved in the p53 pathway. AURKA causes the loss of p53 DNA-binding and transcriptional activation activity through phosphorylation of p53 Ser215 (Liu et al., 2004). Both *in vitro* and *in vivo* experiments have demonstrated that the inhibition of AURKA can induce cellular senescence (Huck et al., 2010). In ovarian cancer, AURKA regulates the p16 pathway through the SOX8-FOXK1 signaling axis, inhibiting cellular senescence and enhancing glucose metabolism, ultimately leading to cisplatin resistance (Sun et al., 2020).

Our study finds that high expression of AURKA gene in high-risk NB is closely related to poor prognosis in children. We speculated that the possible mechanism is related to the inhibition of tumor cellular senescence by p53/p16 signaling pathway, which leads to increased tumor aggressiveness. Additionally, other findings suggest that besides being associated with cellular senescence, the AURKA gene can drive tumor progression through the LIN28B-RAN-AURKA signaling pathway (Schnepp et al., 2015), and it also plays a role in maintaining N-MYC stability in MYCN-amplified NB (Otto et al., 2009). Emerging pre-clinical evidence suggests that inhibitors targeting AURKA, such as MLN8237 (Görgün et al., 2010) and ENMD-2076 (Diamond et al., 2011), could disrupt NB progression and are currently undergoing clinical evaluation. Notably, MLN8237 shows potential for inducing N-myc protein degradation (Brockmann et al., 2013).

CENPA, an H3-related histone variant (Stirpe and Heun, 2023), is integral to centromere assembly (Rosin and Mellone, 2017). Studies have shown that in human primary fibroblasts, reduction of CENPA leads to cellular senescence dependent on the p53 pathway (Maehara et al., 2010).And the elevated expression levels in several malignancies, including hepatocellular carcinoma, renal clear cell carcinoma, breast cancer, and endometrial carcinoma (Rosin and Mellone, 2017; Wang et al., 2021; Liao et al., 2023; Stirpe and Heun, 2023; Li et al., 2024; Wu et al., 2024) have been associated with tumor progression, metastasis, and poor prognosis. Our study confirmed that CENPA is highly expressed in high-risk NB samples and is associated with poor prognosis, suggesting that CENPA may be involved in the negative regulation of cellular senescence in NB through the P53 pathway.

MAD2L1 (Mitotic arrest deficient two like 1) is a component of the spindle assembly checkpoint during mitosis (Yang et al., 2008). In studies related to pulmonary fibrosis, mitochondrial dysfunction caused by inhibition of MAD2L1leads to cellular senescence (Wang et al., 2022). HJURP (Holliday junction recognition protein) is a companion protein of CENP-A during cell division and is involved in the assembly of nucleosome centromere (Foltz et al., 2009). In studies on human primary cellular senescence, Jong-Ik Heo et al. found that knocking down HJURP may induce DNA damage response to activate p53 pathway, leading to cellular senescence (Heo et al., 2013). TACC3 is an important component of mitotic spindles (Gergely, 2002) and a phosphorylation target of AURKA (Aurora a) (LeRoy et al., 2007). In studies related to breast cancer, Schmidt et al. (2010) proposed to regulate the p21 pathway through the Aurora A-TACC3 axis, thereby inhibiting cellular senescence. In our study, MAD2L1, HJURP, and TACC3 were highly expressed in both the training and validation cohorts in high-risk group of NB and were strongly associated with poor outcomes in children.

Based on GEO database, we constructed a prognostic model of five cellular senescence-related genes through LASSO algorithm and established a new risk scoring formula, which was well validated in ArrayExpress database. We reclassified patients into low-risk and high-risk groups according to the median SRS score, finding that the overall survival rate of children in the low-risk group was significantly higher than that in the high-risk group. This indicates that, in addition to the application of COG risk stratification, multi-mode clinical monitoring combined with necessary treatments should be adopted to further improve the survival rate of high-risk NB. Furthermore, the development of highly specific inhibitors targeting the AURKA gene, such as MLN8237 and ENMD-2076, suggests that cellular senescence-related genes could become new targets for NB treatment. Although our prognostic model has been validated using publicly accessible databases, its robustness requires further confirmation via multi-center clinical trials.

### 4.1 Summary

Our investigation revealed five cellular senescence-related genes in NB, from which we constructed prognostic models capable of effectively predicting patients’ outcomes. Notably, among these genes, AURKA and CENPA have emerged as highly promising markers for evaluating cellular senescence. Their significant correlation with NB prognosis underscores their potential as therapeutic targets, providing valuable insights for the advancement of neuroblastoma treatments. This study emphasizes the importance of cellular senescence in NB and lays the foundation for further investigations into targeted therapeutic approaches.

## Data Availability

Publicly available datasets were analyzed in this study. The NB gene expression datasets were downloaded from the Gene Expression Omnibus (GEO) (http://www.ncbi.nlm.nih.gov/geo/) under accession number GSE49710 (Zhang et al., n = 498) and ArrayExpress (https://www.ebi.ac.uk/biostudies/arrayexpress) under accession number E-MTAB-8248 (Christoph et al., n = 223).

## Supplementary material

SUPPLEMENTARY FIGURE S1 GO and KEGG Pathway Enrichment Analysis of SRGs. Visualization of Gene Ontology (GO) and Kyoto Encyclopedia of Genes and Genomes (KEGG) pathway enrichment for the eight SRGs, depicted with a color gradient from green (low ratio) to red (high ratio).

## Abbreviations

NB, neuroblastoma; GEO, Gene Expression Omnibus; DEGs, Differentially Expressed Genes; SRGs, cellular senescence-related genes; OS, Overall survival; SRS, Cellular senescence related signatures; ROC, receiver operating characteristic; KEGG, Kyoto Encyclopedia of Genes and Genome; GO, Gene Ontology; AUC, area under the curve; RT-qPCR, Real-time fluorescence quantitative polymerase chain reaction; BP, biological process; CC, cellular component; MF, molecular function; K-M, Kaplan‒Meier.
