# Supplementary material for: Investigation of differentially expressed genes related to cellular senescence between high-risk and non-high-risk groups in neuroblastoma
Source: Front Cell Dev Biol. 2024 Jul 29;12:1421673. doi: 10.3389/fcell.2024.1421673 (PMC11317289; doi:10.3389/fcell.2024.1421673)
Supplement: Supplementary file 1 [file Table1.DOCX]

**Supplementary Table S1.** Clinical characteristics of enrolled patients in each dataset

|  | **Training cohort** | **Validation cohort** |
| --- | --- | --- |
| **Characteristics** | **GSE49710** | **E-MTAB-8248** |
| **Patients, n** | 498 | 223 |
| **Age (day)** |  |  |
| **Mean** | 899 | 795 |
| **Gender** |  |  |
| **Male** | 287 | - |
| **Female** | 211 | - |
| **MYCN ststus** |  |  |
| **Amplified** | 92 | 46 |
| **Non Amplified** | 401 | 176 |
| **Unknown** | 5 | 1 |
| **High Risk** |  |  |
| **Yes** | 176 | - |
| **No** | 322 | - |
| **INSS Stage** |  |  |
| **1** | 121 | 29 |
| **2** | 78 | 39 |
| **3** | 63 | 36 |
| **4** | 183 | 89 |
| **4S** | 53 | 30 |
| **Survival** |  |  |
| **Alive** | 393 | 181 |
| **Dead** | 105 | 42 |
