# Supplementary figures and images for: Investigation of differentially expressed genes related to cellular senescence between high-risk and non-high-risk groups in neuroblastoma
Source: Front Cell Dev Biol. 2024 Jul 29;12:1421673. doi: 10.3389/fcell.2024.1421673 (PMC11317289; doi:10.3389/fcell.2024.1421673)

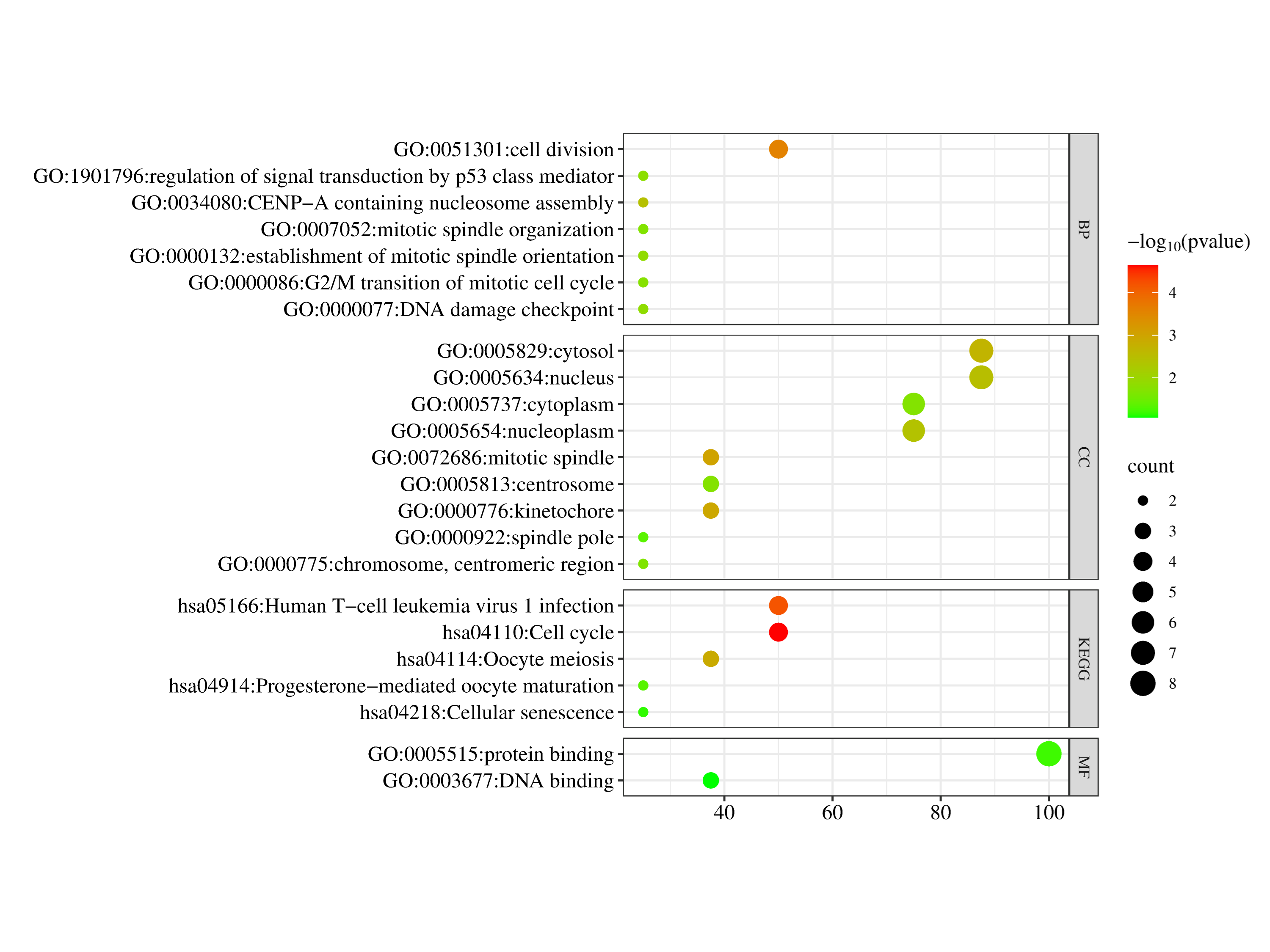

Supplement: Supplementary file 2 [file Image1.TIF]
