# Supplementary material for: Investigation of differentially expressed genes related to cellular senescence between high-risk and non-high-risk groups in neuroblastoma
Source: Front Cell Dev Biol. 2024 Jul 29;12:1421673. doi: 10.3389/fcell.2024.1421673 (PMC11317289; doi:10.3389/fcell.2024.1421673)
Supplement: Supplementary file 3 [file Table2.DOCX]

**Supplementary Table S2.** Genes of cellular senescence

| **Gene Symbol** | **Entrez ID** |
| --- | --- |
| ACLY | 47 |
| AAK1 | 22848 |
| ABI3 | 51225 |
| ADCK5 | 203054 |
| AKR1B1 | 231 |
| AGT | 183 |
| AKT1 | 207 |
| ALOX15B | 247 |
| AR | 367 |
| ARPC1B | 10095 |
| ASF1A | 25842 |
| ASPH | 444 |
| ATF7IP | 55729 |
| ATM | 472 |
| AURKA | 6790 |
| AXL | 558 |
| BAG3 | 9531 |
| BHLHE40 | 8553 |
| BCL6 | 604 |
| BLK | 640 |
| BLVRA | 644 |
| BMI1 | 648 |
| BRAF | 673 |
| BRD7 | 29117 |
| BRCA1 | 672 |
| BTG3 | 10950 |
| C11orf31 | 280636 |
| CAV1 | 857 |
| CBX7 | 23492 |
| CBX8 | 57332 |
| CCND1 | 595 |
| CDK1 | 983 |
| CDK18 | 5129 |
| CDK2AP1 | 8099 |
| CDK6 | 1021 |
| CDK4 | 1019 |
| CDKN1A | 1026 |
| CDKN1C | 1028 |
| CDKN1B | 1027 |
| CDKN2A | 1029 |
| CDKN2AIP | 55602 |
| CDKN2B | 1030 |
| CENPA | 1058 |
| CEBPB | 1051 |
| CHEK1 | 1111 |
| CKB | 1152 |
| CPEB1 | 64506 |
| CSNK1A1 | 1452 |
| CTNNAL1 | 8727 |
| CSNK2A1 | 1457 |
| CXCL1 | 2919 |
| DDB2 | 1643 |
| CYR61 | 3491 |
| DEK | 7913 |
| DGCR8 | 54487 |
| DHCR24 | 1718 |
| DLX2 | 1746 |
| DHX9 | 1660 |
| DPY30 | 84661 |
| DUSP3 | 1845 |
| DUSP16 | 80824 |
| E2F1 | 1869 |
| EHF | 26298 |
| ENDOG | 2021 |
| EPHA3 | 2042 |
| ERRFI1 | 54206 |
| ETS1 | 2113 |
| ETS2 | 2114 |
| EWSR1 | 2130 |
| FASTK | 10922 |
| EZH2 | 2146 |
| FBXO31 | 79791 |
| FOXM1 | 2305 |
| FOS | 2353 |
| FOXO3 | 2309 |
| FXR1 | 8087 |
| G6PD | 2539 |
| GAPDH | 2597 |
| GKN1 | 56287 |
| GATA4 | 2626 |
| GNG11 | 2791 |
| GLB1 | 2720 |
| GRK6 | 2870 |
| HDAC4 | 9759 |
| HDAC1 | 3065 |
| HEPACAM | 220296 |
| HJURP | 55355 |
| HIVEP1 | 3096 |
| HK3 | 3101 |
| HMGB1 | 3146 |
| HRAS | 3265 |
| HSPA5 | 3309 |
| HSPB2 | 3316 |
| ID1 | 3397 |
| ID4 | 3400 |
| IGFBP1 | 3484 |
| IFNG | 3458 |
| IGFBP3 | 3486 |
| IGFBP6 | 3489 |
| IGFBP5 | 3488 |
| IL1A | 3552 |
| IL8 | 3576 |
| ING1 | 3621 |
| ING2 | 3622 |
| IRF3 | 3661 |
| IRF5 | 3663 |
| IRF7 | 3665 |
| ITPK1 | 3705 |
| ITGB4 | 3691 |
| ITPKB | 3707 |
| ITSN2 | 50618 |
| KCNJ12 | 3768 |
| KDM4A | 9682 |
| KDM5B | 10765 |
| KIAA1524 | 57650 |
| KL | 9365 |
| KSR2 | 283455 |
| LATS1 | 9113 |
| LEO1 | 123169 |
| LGALS3 | 3958 |
| LIMA1 | 51474 |
| LIMK1 | 3984 |
| MAGEA2 | 4101 |
| MAGOH | 4116 |
| MAD2L1 | 4085 |
| MAGOHB | 55110 |
| MAP2K1 | 5604 |
| MAP2K3 | 5606 |
| MAP2K2 | 5605 |
| MAP2K6 | 5608 |
| MAP3K6 | 9064 |
| MAP2K7 | 5609 |
| MAP4K1 | 11184 |
| MAP3K7 | 6885 |
| MAPK12 | 6300 |
| MAPKAPK5 | 8550 |
| 5-Mar | 54708 |
| MAPK14 | 1432 |
| MAST1 | 22983 |
| MATK | 4145 |
| MCL1 | 4170 |
| MDH1 | 4190 |
| MCRS1 | 10445 |
| MECP2 | 4204 |
| MOB3A | 126308 |
| MMP9 | 4318 |
| MORC3 | 23515 |
| MORF4 | 10934 |
| MXD4 | 10608 |
| MVK | 4598 |
| MYC | 4609 |
| MYLK | 4638 |
| NADK | 65220 |
| NANOG | 79923 |
| NDRG1 | 10397 |
| NEK1 | 4750 |
| NEK4 | 6787 |
| NEK6 | 10783 |
| NFE2L2 | 4780 |
| NINJ1 | 4814 |
| NOTCH3 | 4854 |
| NOX4 | 50507 |
| NR2E1 | 7101 |
| NTN4 | 59277 |
| NUAK1 | 9891 |
| OTX2 | 5015 |
| P3H1 | 64175 |
| PATZ1 | 23598 |
| PAK4 | 10298 |
| PBRM1 | 55193 |
| PCGF2 | 7703 |
| PDCD10 | 11235 |
| PDIK1L | 149420 |
| PDZD2 | 23037 |
| PDPK1 | 5170 |
| PEBP1 | 5037 |
| PEX19 | 5824 |
| PIAS4 | 51588 |
| PIK3R5 | 23533 |
| PIK3C2A | 5286 |
| PIM1 | 5292 |
| PLA2R1 | 22925 |
| PKM | 5315 |
| PML | 5371 |
| PNPT1 | 87178 |
| PMVK | 10654 |
| POT1 | 25913 |
| POU5F1 | 5460 |
| PPM1B | 5495 |
| PPM1D | 8493 |
| PRMT6 | 55170 |
| PRKCH | 5583 |
| PRKCD | 5580 |
| PROX1 | 5629 |
| PRPF19 | 27339 |
| PSMB5 | 5693 |
| PTRF | 284119 |
| PTTG1 | 9232 |
| PSMD14 | 10213 |
| RAD21 | 5885 |
| RAF1 | 5894 |
| RB1 | 5925 |
| RBP2 | 5948 |
| RBX1 | 9978 |
| RNASEL | 6041 |
| RPS6KA6 | 27330 |
| RSL1D1 | 26156 |
| RUNX1 | 861 |
| RUVBL2 | 10856 |
| SENP1 | 29843 |
| SENP2 | 59343 |
| SENP7 | 57337 |
| SERPINE1 | 5054 |
| SFN | 2810 |
| SIK1 | 150094 |
| SGK1 | 6446 |
| SIN3B | 23309 |
| SIRT1 | 23411 |
| SIRT6 | 51548 |
| SIX1 | 6495 |
| SLC13A3 | 64849 |
| SLC16A7 | 9194 |
| SMARCA4 | 6597 |
| SMG1 | 23049 |
| SMARCB1 | 6598 |
| SMURF2 | 64750 |
| SNAI1 | 6615 |
| SOCS1 | 8651 |
| SOD1 | 6647 |
| SORBS2 | 8470 |
| SOX2 | 6657 |
| SPIN1 | 10927 |
| SOX5 | 6660 |
| SP1 | 6667 |
| SPOP | 8405 |
| SRC | 6714 |
| SREBF1 | 6720 |
| SRSF1 | 6426 |
| STAT5B | 6777 |
| STK32C | 282974 |
| STK40 | 83931 |
| SUPT5H | 6829 |
| SYK | 6850 |
| TACC3 | 10460 |
| TERC | 7012 |
| TBX2 | 6909 |
| TERF2 | 7014 |
| TERT | 7015 |
| TFAP4 | 7023 |
| TFDP1 | 7027 |
| TGFB1I1 | 7041 |
| TLR3 | 7098 |
| TMSB4X | 7114 |
| TNFSF13 | 8741 |
| TNFSF15 | 9966 |
| TOP1 | 7150 |
| TP63 | 8626 |
| TPR | 7175 |
| TP53 | 7157 |
| TRIM28 | 10155 |
| TRPM8 | 79054 |
| TXN | 7295 |
| TXNIP | 10628 |
| UBTD1 | 80019 |
| TYK2 | 7297 |
| VENTX | 27287 |
| USP1 | 7398 |
| VEGFA | 7422 |
| WNT16 | 51384 |
| WNT2 | 7472 |
| WRN | 7486 |
| WT1 | 7490 |
| XAF1 | 54739 |
| WWP1 | 11059 |
| YAP1 | 10413 |
| YPEL3 | 83719 |
| ZFP36 | 7538 |
| ZMAT3 | 64393 |
| ZNF148 | 7707 |
