# Supplementary material for: Investigation of differentially expressed genes related to cellular senescence between high-risk and non-high-risk groups in neuroblastoma
Source: Front Cell Dev Biol. 2024 Jul 29;12:1421673. doi: 10.3389/fcell.2024.1421673 (PMC11317289; doi:10.3389/fcell.2024.1421673)
Supplement: Supplementary file 4 [file Table5.docx]

**Supplementary Table S5.** Univariate Cox analysis of cellular senescence-related genes in GSE49710 cohort

| **Gene_Symbol** | **HR** | **HR.95L** | **HR.95H** | ***P* value** |
| --- | --- | --- | --- | --- |
| TACC3 | 2.9660 | 2.369 | 3.713 | 0.000 |
| CHEK1 | 2.1610 | 1.846 | 2.531 | 0.000 |
| E2F1 | 2.3920 | 1.967 | 2.908 | 0.000 |
| AURKA | 3.0920 | 2.499 | 3.825 | 0.000 |
| MAD2L1 | 2.6340 | 2.158 | 3.215 | 0.000 |
| HJURP | 2.3990 | 1.978 | 2.910 | 0.000 |
| CENPA | 2.4950 | 2.088 | 2.983 | 0.000 |
| PTTG1 | 3.0410 | 2.362 | 3.915 | 0.000 |
