# Supplementary material for: Investigation of differentially expressed genes related to cellular senescence between high-risk and non-high-risk groups in neuroblastoma
Source: Front Cell Dev Biol. 2024 Jul 29;12:1421673. doi: 10.3389/fcell.2024.1421673 (PMC11317289; doi:10.3389/fcell.2024.1421673)
Supplement: Supplementary file 5 [file Table3.DOCX]

**Supplementary Table S3.** DEGs between high-risk and non-high-risk group in GSE49710 cohort

| **Gene Symbol** | **logFC** | ***P* value** | **FDR** |
| --- | --- | --- | --- |
| ERCC6L | 1.665532813 | 1.93962048007839e-37 | 1.92604313671784e-33 |
| PGM2L1 | -1.288094644 | 1.26326997467289e-36 | 6.27213542425092e-33 |
| HIST1H1B | 1.834989922 | 5.6989155670151e-35 | 1.886341052682e-31 |
| PPIL5 | 1.103860708 | 2.93902608991233e-34 | 6.48545090507321e-31 |
| TACC3 | 1.18133867 | 1.08866437502925e-33 | 1.91984167383033e-30 |
| MPST | 1.00338229 | 8.31769031897216e-33 | 8.69417524919932e-30 |
| NEIL3 | 1.726061606 | 1.33648245640901e-32 | 1.10593923267846e-29 |
| UHRF1 | 1.421811347 | 3.34495254653811e-32 | 2.55502913747103e-29 |
| LOC645360 | 1.049457934 | 4.16972219824523e-32 | 2.8911840260778e-29 |
| LOC728688 | 1.388802929 | 1.17610093369367e-31 | 7.07798925550188e-29 |
| WSB1 | -1.017792159 | 4.15895646894817e-31 | 2.06492188683277e-28 |
| GCUD2 | 1.244903479 | 5.1317868742822e-31 | 2.48578749568889e-28 |
| CHAF1A | 1.040869881 | 6.67461093707106e-31 | 3.08273891186584e-28 |
| AURKA | 1.155773566 | 1.31500764780561e-30 | 5.67740258378682e-28 |
| MGC24975 | 1.053097809 | 5.71323570328306e-30 | 1.9562907080552e-27 |
| MGC5457 | -1.326930256 | 6.82383691803146e-30 | 2.27735551840473e-27 |
| PMP22 | -1.393284576 | 7.38753279098978e-30 | 2.37885975629426e-27 |
| FANCI | 1.083867981 | 8.17706773177544e-30 | 2.57772325639778e-27 |
| PLXNC1 | -1.301726955 | 8.60842614326806e-30 | 2.67130223758287e-27 |
| C15orf42 | 1.451241259 | 1.08056886568287e-29 | 3.30155348807103e-27 |
| CENPA | 1.453255854 | 1.41363661265501e-29 | 4.01068901818979e-27 |
| E2F1 | 1.331317662 | 1.55741906594059e-29 | 4.35638628867325e-27 |
| HJURP | 1.267850321 | 1.8139552527433e-29 | 4.93495223554547e-27 |
| SPC24 | 1.438570263 | 6.2176317967073e-29 | 1.47002580336437e-26 |
| CDCA2 | 1.376553324 | 1.0757363306177e-28 | 2.4556463823066e-26 |
| HIST1H2AL | 1.415006618 | 1.81934849192956e-28 | 3.84385755848096e-26 |
| NCAPG | 1.341328247 | 2.13298075657994e-28 | 4.41260394017476e-26 |
| CCNA2 | 1.160108532 | 2.33965104356276e-28 | 4.74137446175066e-26 |
| ATP2B4 | -1.185387705 | 5.1404272984826e-28 | 9.91154234445285e-26 |
| HMG4L | 1.019898197 | 5.33563107103227e-28 | 1.01890031798751e-25 |
| FEN1 | 1.040560541 | 8.824482071184e-28 | 1.6325211034976e-25 |
| UBE2C | 1.32164725 | 1.80827939569407e-27 | 3.12281989552036e-25 |
| CDT1 | 1.202273452 | 1.97215065098413e-27 | 3.26390932737873e-25 |
| KIF4A | 1.105114203 | 2.12899410676485e-27 | 3.4657231934713e-25 |
| TPX2 | 1.092172743 | 2.24579318016391e-27 | 3.59689133532703e-25 |
| BRIP1 | 1.407366547 | 4.26201326595825e-27 | 6.61277995796335e-25 |
| BRCA2 | 1.181037367 | 6.79516135264032e-27 | 9.92293415172329e-25 |
| SPC25 | 1.355454408 | 7.61566356999488e-27 | 1.10399327372335e-24 |
| CEP152 | 1.175230586 | 8.93508649010286e-27 | 1.27662458772261e-24 |
| FAM83D | 1.350313183 | 1.87829727199074e-26 | 2.55499889189974e-24 |
| E2F8 | 1.420877695 | 4.11264339509733e-26 | 5.33837240696947e-24 |
| SLC1A5 | 1.078228294 | 5.52271881965053e-26 | 7.0134716124869e-24 |
| C12orf48 | 1.012648669 | 8.02871409144299e-26 | 9.72257694244255e-24 |
| LOC646993 | 1.172062976 | 8.7429232128567e-26 | 1.03972727549302e-23 |
| TRIM59 | 1.143554341 | 1.67914524202023e-25 | 1.88405788172439e-23 |
| BUB1B | 1.056985838 | 1.91509653891649e-25 | 2.10131587087743e-23 |
| KIF20A | 1.230126554 | 2.65351212440762e-25 | 2.83326617154491e-23 |
| BIRC5 | 1.346756145 | 3.20673506625316e-25 | 3.38754034126531e-23 |
| VSTM2A | -1.561746955 | 3.41392512148851e-25 | 3.58700078046151e-23 |
| SCG2 | -1.268737125 | 3.43167244857848e-25 | 3.58700078046151e-23 |
| GINS2 | 1.135260663 | 3.6686821164656e-25 | 3.76932300674434e-23 |
| DTL | 1.224458519 | 3.6820174386123e-25 | 3.76932300674434e-23 |
| CDC6 | 1.078595235 | 4.36066923696221e-25 | 4.37388338616512e-23 |
| MLF1IP | 1.158702822 | 4.44552872573919e-25 | 4.43659298960705e-23 |
| MAP7 | -1.167513822 | 4.84657482082148e-25 | 4.74152590844899e-23 |
| MND1 | 1.170320789 | 5.25563500357628e-25 | 5.09155664248902e-23 |
| C18orf24 | 1.307747551 | 5.39622731689677e-25 | 5.20238225794028e-23 |
| ZNF695 | 1.377245809 | 8.30550122988327e-25 | 7.74400255518693e-23 |
| CCNB2 | 1.239842983 | 8.44228967120706e-25 | 7.83476041449403e-23 |
| MYBL2 | 1.078162879 | 8.634464235097e-25 | 7.97583533530355e-23 |
| DLGAP5 | 1.263810805 | 9.33942399385594e-25 | 8.51683475415496e-23 |
| CCNB1 | 1.038289665 | 1.3033764540897e-24 | 1.13530949027287e-22 |
| CHD5 | -1.442955999 | 1.34231208616309e-24 | 1.15403974161034e-22 |
| CEP55 | 1.160014639 | 1.39969981607736e-24 | 1.19819130807312e-22 |
| LOC127295 | 1.05484852 | 2.18630013450133e-24 | 1.78682801116035e-22 |
| BLM | 1.025311439 | 2.50265507589804e-24 | 2.00331854190791e-22 |
| CDCA5 | 1.16211865 | 2.86139365746866e-24 | 2.24613747183113e-22 |
| PTTG1 | 1.086373813 | 3.2904294280878e-24 | 2.56266386046367e-22 |
| C9orf140 | 1.055126909 | 3.3208212186834e-24 | 2.57623083605673e-22 |
| C19orf40 | 1.111368881 | 3.74972063848881e-24 | 2.87526841237018e-22 |
| MKI67 | 1.316468038 | 3.77603785789097e-24 | 2.88431199452749e-22 |
| PTTG2 | 1.083162052 | 4.17591295865992e-24 | 3.15896080092812e-22 |
| CENPE | 1.019528598 | 4.43265541546182e-24 | 3.30949385530345e-22 |
| MAD2L1 | 1.129448073 | 4.95246722508739e-24 | 3.64281478111983e-22 |
| RAD51 | 1.089667095 | 5.96802113532306e-24 | 4.23892208197751e-22 |
| CDKN3 | 1.076466267 | 6.06562461124036e-24 | 4.28695034801543e-22 |
| EXO1 | 1.335132447 | 1.13805950056705e-23 | 7.61005443813519e-22 |
| FBXO43 | 1.318750567 | 1.53855513824572e-23 | 9.98552452469278e-22 |
| HIST1H1C | 1.128359731 | 1.55607098461862e-23 | 1.00570070529883e-21 |
| AMPH | -1.15766087 | 1.59426601353903e-23 | 1.02466417569208e-21 |
| KIF18A | 1.297626081 | 1.76477805760448e-23 | 1.11265054679444e-21 |
| KIAA0101 | 1.085774789 | 2.53190190673446e-23 | 1.55196209468353e-21 |
| CENPN | 1.073272753 | 3.18884884838761e-23 | 1.92494036866194e-21 |
| CHEK1 | 1.179041607 | 3.92691455558986e-23 | 2.30735275366907e-21 |
| NCAPH | 1.08090771 | 5.57292838646652e-23 | 3.16223879300643e-21 |
| LOC146909 | 1.261867188 | 5.87931064373575e-23 | 3.28910167280541e-21 |
| MCM10 | 1.040783513 | 8.88185106320027e-23 | 4.84597698118564e-21 |
| GSG2 | 1.117458235 | 9.70995416917993e-23 | 5.2544874604881e-21 |
| KIF15 | 1.099757564 | 1.22803653762633e-22 | 6.43504106523981e-21 |
| SLC22A4 | -1.061669779 | 1.44178936423544e-22 | 7.37988061178243e-21 |
| CLSPN | 1.103512329 | 1.56093847851943e-22 | 7.88810131893025e-21 |
| CDC45L | 1.176855671 | 2.2880271212715e-22 | 1.14171403589075e-20 |
| C13orf3 | 1.076527212 | 2.78398031205179e-22 | 1.3584729483378e-20 |
| BUB1 | 1.047436818 | 3.79255982995001e-22 | 1.81932942567167e-20 |
| LOC653801 | 1.015708016 | 4.96801550562755e-22 | 2.32699971560762e-20 |
| HS6ST3 | -1.651970055 | 5.9714174245101e-22 | 2.73254262789794e-20 |
| TK1 | 1.031542483 | 1.65903969109498e-21 | 6.7379403405207e-20 |
| LOC649056 | 1.165856521 | 1.92089005252899e-21 | 7.59937777753499e-20 |
| CDCA7 | 1.029294465 | 2.10131634167635e-21 | 8.2801870130342e-20 |
| AURKB | 1.150728097 | 2.29598405862835e-21 | 8.94083204007039e-20 |
| CENPF | 1.116157526 | 2.52013581864548e-21 | 9.79450046150669e-20 |
| PLK4 | 1.000470781 | 2.59559907678178e-21 | 1.00484595837985e-19 |
| NTRK1 | -2.353056272 | 3.48671109071864e-21 | 1.31646544223711e-19 |
| SLC22A16 | 1.108363651 | 5.19820028189727e-21 | 1.89077394869011e-19 |
| LOC339192 | -1.340930277 | 7.72081643305747e-21 | 2.70432829560003e-19 |
| RAB3B | -1.456838834 | 8.39533623416166e-21 | 2.93025268208173e-19 |
| ABCC4 | 1.325738348 | 9.25530928264627e-21 | 3.2078611230952e-19 |
| TRPV2 | -1.17064707 | 9.43804356016858e-21 | 3.26549730147993e-19 |
| CD163L1 | -1.084176985 | 1.14090370339053e-20 | 3.87322180330527e-19 |
| RHBG | 1.612029984 | 2.01356779992934e-20 | 6.53422491937855e-19 |
| ASPM | 1.109902041 | 2.07207890779858e-20 | 6.70219659753742e-19 |
| CDH9 | -1.342779915 | 2.10201959420441e-20 | 6.77696576962657e-19 |
| KLRG2 | 1.215485107 | 2.37352628268539e-20 | 7.59069758037549e-19 |
| DYDC2 | 1.250312187 | 3.43257008375668e-20 | 1.07018590052445e-18 |
| HPCAL4 | -1.41414463 | 3.73481983623196e-20 | 1.15896128043073e-18 |
| GTSE1 | 1.040286259 | 4.12795832782021e-20 | 1.26905963452801e-18 |
| PRUNE2 | -1.183594172 | 4.64676317402877e-20 | 1.40892697154521e-18 |
| XKR4 | -1.313400426 | 5.23839723242773e-20 | 1.57389665712579e-18 |
| E2F7 | 1.006826001 | 5.8757778854698e-20 | 1.74428921981211e-18 |
| ESCO2 | 1.106727821 | 7.23046675825016e-20 | 2.12421700915456e-18 |
| CDC25C | 1.012063515 | 7.70930826130685e-20 | 2.25488751207002e-18 |
| TEX15 | 1.610861293 | 1.51948710003236e-19 | 4.18543880813351e-18 |
| NRCAM | -1.03259796 | 1.54754853117763e-19 | 4.2568301702476e-18 |
| PCOLCE2 | 1.945050016 | 1.6148590874113e-19 | 4.42971014861717e-18 |
| NUF2 | 1.032112533 | 2.09436122232421e-19 | 5.62841865701744e-18 |
| TMEFF2 | -1.438481485 | 2.80057532511959e-19 | 7.30872877225689e-18 |
| ADCY1 | -1.033565884 | 3.93048529784704e-19 | 9.90602005269572e-18 |
| KIRREL3 | -1.436500512 | 3.97084099871445e-19 | 9.99504464315196e-18 |
| CDH12 | -1.632510661 | 6.42905795821354e-19 | 1.55708647622099e-17 |
| NEK2 | 1.186597701 | 6.54095604489022e-19 | 1.58033317580924e-17 |
| HOXC10 | -1.215934315 | 6.82543150286026e-19 | 1.64317203541375e-17 |
| DNER | -1.322379123 | 9.81119926247791e-19 | 2.30135259008103e-17 |
| KLF15 | 1.247653908 | 1.12262896067935e-18 | 2.60765042796396e-17 |
| ABCA4 | 1.01577839 | 1.5513122695176e-18 | 3.50102973552494e-17 |
| PLCD4 | -1.224232457 | 1.6691023441564e-18 | 3.75831888378074e-17 |
| MYOZ3 | 1.10644858 | 1.99792097113129e-18 | 4.4234905782238e-17 |
| CASC5 | 1.017979247 | 2.75585242261093e-18 | 5.97502501234205e-17 |
| C5orf4 | -1.072798656 | 2.99006409806763e-18 | 6.45463836821991e-17 |
| MED12L | 1.066291066 | 5.33403125503354e-18 | 1.09322869685207e-16 |
| SV2B | -1.593513982 | 6.4356624677285e-18 | 1.30821142895689e-16 |
| PHGDH | 1.330577766 | 1.02867235756257e-17 | 2.01871867798347e-16 |
| E2F2 | 1.003650711 | 1.05421225974167e-17 | 2.06679718444912e-16 |
| PTPRH | -1.286039053 | 1.5095717883273e-17 | 2.88270151117116e-16 |
| IGSF9 | 1.019021202 | 1.60382725823507e-17 | 3.0480391721099e-16 |
| NPPC | 1.106572462 | 1.82233627250768e-17 | 3.42835490904399e-16 |
| RRM2 | 1.076428957 | 2.2681964979621e-17 | 4.20208791506784e-16 |
| FLNC | 1.067539239 | 2.30626381681945e-17 | 4.26612314587237e-16 |
| CABP7 | 1.672473229 | 2.32703446632885e-17 | 4.29906088384103e-16 |
| NXPH1 | -1.156034186 | 2.64909961780007e-17 | 4.80906018368459e-16 |
| PKIB | -1.522260467 | 2.73190178644248e-17 | 4.94130869569651e-16 |
| ZNF367 | 1.078356128 | 2.98903924075013e-17 | 5.37702167765377e-16 |
| S100B | -1.292511352 | 3.24106470185161e-17 | 5.79365841393097e-16 |
| CGB1 | 1.581253657 | 4.58597998423171e-17 | 8.01033970860526e-16 |
| DRD1IP | -1.242347151 | 4.68507493839591e-17 | 8.14759967395297e-16 |
| TRIM54 | 1.416598387 | 4.82114754233893e-17 | 8.34768876990856e-16 |
| LOC100127887 | -1.823865735 | 4.96345804072315e-17 | 8.55337001674977e-16 |
| IL24 | -1.206268032 | 5.6868717191867e-17 | 9.74471719957272e-16 |
| MATN2 | -1.0030964 | 6.69797084119859e-17 | 1.13017587855738e-15 |
| FAM70A | -1.389419424 | 6.75502204640001e-17 | 1.13883478643043e-15 |
| ADAM7 | -1.118967299 | 9.31431819705817e-17 | 1.52751741860921e-15 |
| RHBDL3 | -1.059557965 | 9.71045932776811e-17 | 1.58593521586739e-15 |
| FAM111B | 1.145755413 | 1.35688612305624e-16 | 2.16447858665839e-15 |
| MDGA1 | -1.406542119 | 1.48332529901678e-16 | 2.34544907949628e-15 |
| SLCO4A1 | 1.801410541 | 1.69171250757365e-16 | 2.65802297471619e-15 |
| NUP62CL | 1.065669279 | 1.81922184409039e-16 | 2.84262358958577e-15 |
| CRYGD | 1.151768475 | 2.03834651317032e-16 | 3.1675713420628e-15 |
| PLXNA4 | -1.38741229 | 2.29441484496883e-16 | 3.53507205749271e-15 |
| SYT13 | -1.178683361 | 3.93415683979193e-16 | 5.89233445235805e-15 |
| KIF6 | 1.178686531 | 4.10382910178559e-16 | 6.11877221932897e-15 |
| NTS | 1.59653455 | 4.2013173717427e-16 | 6.25942708198124e-15 |
| ENTPD3 | -1.428227975 | 4.5535393431727e-16 | 6.74875308622461e-15 |
| SCN7A | -1.155862253 | 5.39381019109416e-16 | 7.92315609431434e-15 |
| AKR1C1 | -1.025812762 | 7.24848833534417e-16 | 1.04315201695605e-14 |
| SOX6 | -1.372155811 | 7.59762883556792e-16 | 1.08709588382117e-14 |
| OR4K17 | -1.053417271 | 9.48417480717592e-16 | 1.33490936690655e-14 |
| RGS9 | -1.514975945 | 1.00741040168614e-15 | 1.4109429180174e-14 |
| NAV3 | -1.278731549 | 1.19495554335623e-15 | 1.64689917356382e-14 |
| RP4-662A9.2 | -1.035269431 | 1.33437514815534e-15 | 1.82637425515955e-14 |
| CDH10 | -1.763293543 | 1.42577985905925e-15 | 1.94611601380871e-14 |
| RLN2 | 1.065897594 | 1.70302053141259e-15 | 2.28262379474085e-14 |
| IL18 | -1.069900501 | 2.03907779337991e-15 | 2.70334345637684e-14 |
| PTGIS | 1.107025006 | 2.87697060592998e-15 | 3.74666467106685e-14 |
| EPB41L3 | -1.172247316 | 3.03552021008106e-15 | 3.94022427269345e-14 |
| TRPM3 | -1.273577355 | 3.1180480507798e-15 | 4.03679493406042e-14 |
| CA10 | -1.303536327 | 4.40385871550176e-15 | 5.54953261991529e-14 |
| FLJ39502 | -1.051805914 | 4.43962940570523e-15 | 5.58558467474375e-14 |
| LOC648969 | 1.012770566 | 5.21299529170177e-15 | 6.48684752463642e-14 |
| BAIAP3 | -1.295861251 | 5.30280614850493e-15 | 6.57799688377938e-14 |
| AMIGO2 | -1.722415244 | 6.21426708357357e-15 | 7.62147634067224e-14 |
| CAPS2 | -1.001837924 | 6.89786407878904e-15 | 8.40439144814419e-14 |
| C11orf87 | -1.130285157 | 9.00447953425676e-15 | 1.07211608843129e-13 |
| ADCYAP1 | -1.825888467 | 9.59005085622159e-15 | 1.13706513435559e-13 |
| CGNL1 | -1.031407323 | 1.01391585024536e-14 | 1.19788035608999e-13 |
| OR51G1 | -1.04100471 | 1.04785702195965e-14 | 1.23504097662425e-13 |
| DOCK3 | -1.346013795 | 1.1208176782407e-14 | 1.31401647519837e-13 |
| RP13-102H20.1 | -2.065115645 | 1.19163932425257e-14 | 1.39375482801273e-13 |
| HK2 | 1.317177673 | 1.5117219416094e-14 | 1.73542183585912e-13 |
| TAS2R41 | -1.099503667 | 1.66830897329581e-14 | 1.8976297943674e-13 |
| FOXG1B | -1.103735579 | 1.82189311028288e-14 | 2.06239381113001e-13 |
| POF1B | -1.855840344 | 1.88019293928928e-14 | 2.1216268053571e-13 |
| UNQ6125 | -1.022394618 | 2.2293142056287e-14 | 2.47895745373942e-13 |
| LUZPP1 | -1.007768071 | 2.24802585728422e-14 | 2.49417840925501e-13 |
| MC4R | -1.156070142 | 3.28589854550354e-14 | 3.55048667647989e-13 |
| NSUN7 | 1.206188415 | 3.34673266913211e-14 | 3.61032649695621e-13 |
| EPHA5 | -1.451839233 | 3.87041022723455e-14 | 4.12594455785713e-13 |
| SLC18A2 | -1.359799978 | 4.36698596333724e-14 | 4.62551153236681e-13 |
| NCAN | 1.610298676 | 4.50706864894784e-14 | 4.76626109521321e-13 |
| DIRAS3 | -1.648479824 | 5.14986428864799e-14 | 5.38863565714168e-13 |
| CLCA2 | -1.042147625 | 6.064826423592e-14 | 6.29625994629049e-13 |
| SHISA3 | 1.207226748 | 8.32775087932809e-14 | 8.43391802465354e-13 |
| DDC | -1.091751832 | 8.38413034389918e-14 | 8.4823651874599e-13 |
| UNC5D | -1.195888281 | 8.87138344286396e-14 | 8.94343528808519e-13 |
| LOC388002 | -1.838815459 | 1.16855723585573e-13 | 1.15633017957622e-12 |
| EFS | 1.01725438 | 1.22422875120478e-13 | 1.20720868912249e-12 |
| KIAA0367 | -1.175958608 | 1.58053996403391e-13 | 1.53269158621648e-12 |
| LOC399947 | -1.168137741 | 1.83398638504413e-13 | 1.76553415448262e-12 |
| KIRREL2 | 1.271823597 | 2.01656444726471e-13 | 1.93473284650615e-12 |
| DUXAP10 | 1.569883619 | 2.07400049498734e-13 | 1.98122413806871e-12 |
| KIAA0319 | -1.01420237 | 2.19383753903672e-13 | 2.08766715502009e-12 |
| PRPH | -1.036220673 | 2.33482556725746e-13 | 2.20912986020644e-12 |
| LRGUK | -1.135630807 | 3.13727588083149e-13 | 2.91287045317033e-12 |
| GRID2 | -1.125982434 | 3.95226128595812e-13 | 3.62047551379743e-12 |
| ANKFN1 | -1.280958805 | 4.04261174312891e-13 | 3.6981238700387e-12 |
| HOXC9 | -1.274164778 | 4.40170821344758e-13 | 3.99899017013124e-12 |
| ITGAD | -1.279152922 | 5.28616634754043e-13 | 4.70776967094856e-12 |
| KRT27 | -1.009237168 | 6.66008275264944e-13 | 5.82553775846342e-12 |
| TTC39A | -1.01129805 | 9.2682386276509e-13 | 7.9545038524264e-12 |
| FAM19A1 | -1.664191513 | 1.08695647469929e-12 | 9.22913877192303e-12 |
| TMEM16D | -1.122331462 | 1.17503430946421e-12 | 9.95146327759456e-12 |
| FOXD1 | -1.166796627 | 1.19983987410012e-12 | 1.0148560434254e-11 |
| IRX5 | 1.445802429 | 1.26552311060078e-12 | 1.06542132159947e-11 |
| C8orf79 | -1.122221322 | 1.34686898959478e-12 | 1.1291185366548e-11 |
| PCSK1 | -1.128768437 | 1.48350596669239e-12 | 1.2353219496231e-11 |
| HPR | 1.447765755 | 1.52286456327738e-12 | 1.26703352436903e-11 |
| KIAA1086 | -1.060943242 | 1.87991174783235e-12 | 1.54340832211453e-11 |
| PTPRZ1 | -1.427673891 | 1.89029292318332e-12 | 1.5512899774554e-11 |
| FLJ14712 | -1.096433128 | 2.10396118147532e-12 | 1.71529840164614e-11 |
| OR4N4 | 1.000766863 | 2.1546186854213e-12 | 1.75515697672137e-11 |
| C1orf173 | -1.119819319 | 2.25983188622258e-12 | 1.8348430605225e-11 |
| NMU | 1.546436948 | 2.38552855522171e-12 | 1.93137371001644e-11 |
| STK33 | 1.204667278 | 3.40221643900375e-12 | 2.70922287404228e-11 |
| PTHR2 | 1.396039504 | 3.46390552556941e-12 | 2.75503258861868e-11 |
| ATP1A2 | -1.336065304 | 3.69401897853649e-12 | 2.93101146279403e-11 |
| PPP2R2C | -1.111201985 | 3.86431557839444e-12 | 3.05514758705866e-11 |
| DGKB | -1.296452626 | 3.94491445140546e-12 | 3.11020250118747e-11 |
| DLX6 | 1.093417922 | 4.30808596017326e-12 | 3.37376132370035e-11 |
| LOC340286 | 1.047274448 | 4.9564368783194e-12 | 3.84210914923588e-11 |
| TMEM163 | -1.121387198 | 5.29126688632412e-12 | 4.08095380048144e-11 |
| DSCR8 | 1.53628598 | 5.93296080302326e-12 | 4.54936685513676e-11 |
| CBLN1 | 1.006746264 | 6.24523676612321e-12 | 4.76856601980803e-11 |
| PLXNA4B | -1.030815816 | 6.50729297587663e-12 | 4.9515263793452e-11 |
| NPW | 1.05533212 | 6.95667999268801e-12 | 5.26122104549825e-11 |
| ECEL1 | -1.455616328 | 7.01629345032485e-12 | 5.30025058666609e-11 |
| GPR128 | -1.09668961 | 7.26930859027649e-12 | 5.47887926386684e-11 |
| KNDC1 | -1.284063196 | 7.31214199407686e-12 | 5.50698293524333e-11 |
| CD86 | 1.739151269 | 8.18493274939108e-12 | 6.12713020742204e-11 |
| CLEC4E | -1.290879827 | 8.90490377260528e-12 | 6.63607463129234e-11 |
| ARPP-21 | -1.25870622 | 9.81237117583266e-12 | 7.26056972995665e-11 |
| FLJ39632 | 1.218955849 | 1.18186669850434e-11 | 8.56637687310077e-11 |
| RPA4 | 1.02698025 | 1.27565193996457e-11 | 9.19580672511667e-11 |
| BTC | 1.005420188 | 1.30083206387823e-11 | 9.36372772331342e-11 |
| ST6GAL2 | 1.078337065 | 1.72977686152104e-11 | 1.22559288154862e-10 |
| FMO3 | -1.029074972 | 2.15146164377133e-11 | 1.50503797975691e-10 |
| RGS5 | -1.033389049 | 2.28777686893959e-11 | 1.59310128391095e-10 |
| PDIA2 | -1.06615251 | 2.33350999190196e-11 | 1.62324022554021e-10 |
| HS3ST5 | -1.208030346 | 2.53094653651922e-11 | 1.75137972875511e-10 |
| FGF10 | 1.186842779 | 2.60017495941609e-11 | 1.79615564153056e-10 |
| KCNMB2 | -1.061075601 | 3.59671408667544e-11 | 2.4429118249444e-10 |
| DLX5 | 1.253737191 | 3.73746676531904e-11 | 2.52813657899305e-10 |
| CDH18 | -1.302362784 | 3.84755707612439e-11 | 2.59818033090209e-10 |
| MGC16291 | 1.560543402 | 4.21122656847526e-11 | 2.83220317134841e-10 |
| DYNC1I1 | -1.080793737 | 4.40808005515742e-11 | 2.95458892660905e-10 |
| AIM1L | 1.269417646 | 4.97084306958491e-11 | 3.30170379136978e-10 |
| FAM19A5 | 1.065728613 | 5.40477831562693e-11 | 3.56252563386495e-10 |
| HP | 1.190002342 | 5.44766773265742e-11 | 3.58722417674325e-10 |
| CREG2 | -1.072812039 | 6.12870925748492e-11 | 4.00645707220706e-10 |
| MGC39584 | 1.09442687 | 7.03694950757635e-11 | 4.55075927126233e-10 |
| SLC27A2 | 1.15237568 | 8.02364409499509e-11 | 5.13699457532567e-10 |
| LOC643401 | 1.194157538 | 8.72767355142926e-11 | 5.5519409587247e-10 |
| SHC3 | 1.079611577 | 1.00085134974195e-10 | 6.31614483821896e-10 |
| RBP4 | 1.626438468 | 1.10064647275882e-10 | 6.90642620821177e-10 |
| SLC18A1 | -1.202852541 | 1.25452649631958e-10 | 7.82257338050454e-10 |
| COL20A1 | -1.160526767 | 1.34209554986779e-10 | 8.33198425144554e-10 |
| GRIN3A | -1.379016522 | 1.63725764463243e-10 | 1.00234083916153e-09 |
| GABRA5 | 1.067854937 | 1.75898194568844e-10 | 1.06928011758103e-09 |
| ADCY2 | -1.208084936 | 2.1762042200466e-10 | 1.30022309898091e-09 |
| CCBE1 | -1.004201171 | 2.33049332264421e-10 | 1.38615146414238e-09 |
| UNQ6975 | 1.228970841 | 2.65682731728754e-10 | 1.57084223046534e-09 |
| LRRTM4 | -1.322873362 | 2.83126980297178e-10 | 1.66614507091556e-09 |
| LY6H | 1.405981507 | 4.30429821011784e-10 | 2.47204634045519e-09 |
| STEAP2 | 1.192105961 | 4.40565619358486e-10 | 2.52514666679929e-09 |
| ADRB2 | -1.184828478 | 4.79471636643098e-10 | 2.73550896401377e-09 |
| MYOT | -1.262504213 | 5.86330002472383e-10 | 3.30247131284785e-09 |
| ASCL1 | 1.119545366 | 6.28148689090343e-10 | 3.51706596147003e-09 |
| TFAP2B | -1.366192654 | 6.75769437910405e-10 | 3.76459496126245e-09 |
| POU4F2 | -1.274007041 | 8.90273511093559e-10 | 4.88555731702627e-09 |
| SYNPR | -1.067401844 | 1.11550661455626e-09 | 6.05134153648928e-09 |
| PCDH20 | -1.046201623 | 1.28416437588988e-09 | 6.89656692946812e-09 |
| C1orf67 | 1.214513467 | 1.9916492064376e-09 | 1.03220650417147e-08 |
| ANKRD43 | -1.368580157 | 2.43048268636872e-09 | 1.24149655738896e-08 |
| SLC30A3 | 1.163643842 | 2.50346037189099e-09 | 1.27516601656207e-08 |
| LMO3 | 1.462700755 | 2.9593720927697e-09 | 1.48770639826011e-08 |
| CUX2 | 1.378426196 | 3.00290291807763e-09 | 1.50828659466418e-08 |
| GCGR | -1.265628688 | 3.11179360175609e-09 | 1.55902660203124e-08 |
| LRRC3B | -1.030976868 | 4.03845714550871e-09 | 1.98819432101644e-08 |
| NAP1L2 | -1.195791434 | 4.30930881203232e-09 | 2.11263571974727e-08 |
| CTAG1A | -1.771219864 | 4.61249450977483e-09 | 2.25071599420462e-08 |
| ALB | 1.296163038 | 5.81093060668511e-09 | 2.77950582487395e-08 |
| KRT19 | -1.36028519 | 6.78397200451667e-09 | 3.21167303956379e-08 |
| LIX1 | 1.033377785 | 7.01187157832485e-09 | 3.31009673272003e-08 |
| KCTD8 | 1.237659316 | 8.49274530222138e-09 | 3.96301507758733e-08 |
| PTGDS | -1.145967936 | 9.08693726253496e-09 | 4.21749413493677e-08 |
| ZNF560 | 1.041519521 | 1.75936299641954e-08 | 7.83078196075574e-08 |
| LOC286382 | -1.050673984 | 1.99068991766813e-08 | 8.79143912939494e-08 |
| LOC253012 | -1.013547088 | 2.54367646167396e-08 | 1.10348218717442e-07 |
| FAM112B | 1.104273955 | 2.70964188952386e-08 | 1.17136900315779e-07 |
| PCSK2 | -1.165026626 | 2.94341074684346e-08 | 1.26446327995482e-07 |
| FABP1 | 1.202028323 | 5.91767105203755e-08 | 2.41870646415859e-07 |
| LRRC2 | 1.008519894 | 7.60934422894545e-08 | 3.05543017361214e-07 |
| CTAG2 | -1.626370192 | 7.75170290074864e-08 | 3.10631193722494e-07 |
| PRG4 | 1.099045721 | 9.69227721600334e-08 | 3.82149345860287e-07 |
| FSTL5 | -1.269115958 | 9.74342259590762e-08 | 3.83861084615603e-07 |
| CSAG1 | -1.034647744 | 9.8144678459945e-08 | 3.86276915222851e-07 |
| LOC402505 | -1.525340391 | 1.21136264389215e-07 | 4.68138978550265e-07 |
| NR4A2 | -1.021549243 | 1.31063816120519e-07 | 5.03662420308342e-07 |
| AMBP | 1.19020306 | 1.43407331508856e-07 | 5.47389891171608e-07 |
| HBD | 1.021956988 | 1.46133477471863e-07 | 5.5683247555472e-07 |
| CDH19 | -1.46112918 | 1.72615447666616e-07 | 6.49952389773478e-07 |
| PLP1 | -1.248818221 | 2.69123477945721e-07 | 9.80875806937425e-07 |
| TM4SF4 | 1.065100204 | 3.01293151647351e-07 | 1.08932859852838e-06 |
| NKX6-2 | 1.096389361 | 3.32634601475081e-07 | 1.19546203135996e-06 |
| KLHL1 | -1.060100302 | 5.93886223396413e-07 | 2.04023186242047e-06 |
| KCNJ6 | 1.116269584 | 6.38221931108556e-07 | 2.18159854592357e-06 |
| VSNL1 | -1.128750171 | 9.78849460004302e-07 | 3.25245947393098e-06 |
| RORB | -1.00275268 | 1.29609670390113e-06 | 4.21120277552939e-06 |
| LEMD1 | -1.005093105 | 1.5297366613969e-06 | 4.91594985361528e-06 |
| ANGPTL7 | -1.158627566 | 1.68253089685435e-06 | 5.36701953285054e-06 |
| IL28RA | 1.030527235 | 1.76848372290305e-06 | 5.61504184442118e-06 |
| PLCXD3 | -1.378040868 | 2.94837143951201e-06 | 8.98077558109025e-06 |
| IL7 | -1.009858673 | 8.65607674542035e-06 | 2.44432936391367e-05 |
| IGF2BP1 | 1.275979848 | 1.29122639378606e-05 | 3.54587336567909e-05 |
| ABCA12 | 1.037453618 | 1.65800299852703e-05 | 4.48182109034256e-05 |
| CCL19 | -1.113115536 | 3.13913189013751e-05 | 8.06404854975178e-05 |
| EYA4 | -1.14043638 | 3.54009834021945e-05 | 9.02984241417395e-05 |
| C7orf16 | -1.109409475 | 4.15911886743741e-05 | 0.000104796 |
| MAGEA2B | -1.041715357 | 0.000761113 | 0.001546522 |
