# Supplementary material for: Investigation of differentially expressed genes related to cellular senescence between high-risk and non-high-risk groups in neuroblastoma
Source: Front Cell Dev Biol. 2024 Jul 29;12:1421673. doi: 10.3389/fcell.2024.1421673 (PMC11317289; doi:10.3389/fcell.2024.1421673)
Supplement: Supplementary file 6 [file Table4.docx]

**Supplementary Table S4.** Cellular senescence-related genes in GSE49710 cohort

| **Gene Symbol** | **logFC** | ***P* value** | **FDR** |
| --- | --- | --- | --- |
| TACC3 | 1.18133867 | 1.08866437502925e-33 | 1.91984167383033e-30 |
| CHEK1 | 1.179041607 | 3.92691455558986e-23 | 2.30735275366907e-21 |
| E2F1 | 1.331317662 | 1.55741906594059e-29 | 4.35638628867325e-27 |
| AURKA | 1.155773566 | 1.31500764780561e-30 | 5.67740258378682e-28 |
| MAD2L1 | 1.129448073 | 4.95246722508739e-24 | 3.64281478111983e-22 |
| HJURP | 1.267850321 | 1.8139552527433e-29 | 4.93495223554547e-27 |
| CENPA | 1.453255854 | 1.41363661265501e-29 | 4.01068901818979e-27 |
| PTTG1 | 1.086373813 | 3.2904294280878e-24 | 2.56266386046367e-22 |
